# Predictive SNPs for β^0^-thalassemia/HbE disease severity

**DOI:** 10.1038/s41598-021-89641-2

**Published:** 2021-05-14

**Authors:** Thongperm Munkongdee, Sissades Tongsima, Chumpol Ngamphiw, Pongsakorn Wangkumhang, Chayanon Peerapittayamongkol, Hafizah Binti Hashim, Suthat Fucharoen, Saovaros Svasti

**Affiliations:** 1 Department of Biochemistry, Faculty of Medicine Siriraj Hospital, Mahidol University, Bangkok, Thailand; 2 Thalassemia Research Center, Institute of Molecular Biosciences, Mahidol University, Nakhon Pathom, Thailand; 3 National Biobank of Thailand (NBT), National Science and Technology Development Agency (NSTDA), Pathum Thani, Thailand; 4 Pathology Department, Hospital Sultanah Bahiyah, Kedah, Malaysia; 5 Department of Biochemistry, Faculty of Science, Mahidol University, Bangkok, Thailand

**Keywords:** Predictive markers, Predictive markers, Genetics research, Molecular medicine

## Abstract

β-Thalassemia/HbE disease has a wide spectrum of clinical phenotypes ranging from asymptomatic to dependent on regular blood transfusions. Ability to predict disease severity is helpful for clinical management and treatment decision making. A thalassemia severity score has been developed from Mediterranean β-thalassemia patients. However, different ethnic groups may have different allele frequency and linkage disequilibrium structures. Here, Thai β^0^-thalassemia/HbE disease genome-wild association studies (GWAS) data of 487 patients were analyzed by SNP interaction prioritization algorithm, interacting Loci (iLoci), to find predictive SNPs for disease severity. Three SNPs from two SNP interaction pairs associated with disease severity were identifies. The three-SNP disease severity risk score composed of rs766432 in *BCL11A*, rs9399137 in *HBS1L-MYB* and rs72872548 in *HBE1* showed more than 85% specificity and 75% accuracy. The three-SNP predictive score was then validated in two independent cohorts of Thai and Malaysian β^0^-thalassemia/HbE patients with comparable specificity and accuracy. The SNP risk score could be used for prediction of clinical severity for Southeast Asia β^0^-thalassemia/HbE population.

## Introduction

Thalassemia, an inherited red blood cell disorder, presents a significant health problem worldwide. β-Thalassemia caused by defects on β-globin gene results in reduction or absence of β-globin chain synthesis. Co-inheritance of β^0^-thalassemia with hemoglobin (Hb) E resulting in β^0^-thalassemia/HbE genotype, the most prevalent β-thalassemia diseases in Southeast Asia including Thailand and Malaysia. In contrast to β-thalassemia major patients, carrying homozygous or compound heterozygous β^0^-thalassemia mutations, who have a severe phenotype of β-thalassemia disease and requiring regular blood transfusions, β^0^-thalassemia/HbE disease has a very heterogeneous clinical symptom. Although carrying the same β-thalassemia mutation, the patients have a remarkable variability in disease severity. The average Hb levels were 7.7 g/dL with a range from 3 to 13 g/dL^1^. The mild and moderate symptoms usually have no need for regular blood transfusions. Whereas severe symptoms have marked anemia, hepatosplenomegaly, heavy iron overload and required regular blood transfusions similar to β-thalassemia major patients.

Genetic factors are involved in determining the β-thalassemia clinical variability. As pathophysiological changes in β-thalassemia patients are predominantly determined by the amount of excess α-globin chains, the factor that affects the imbalance between α- and β-globin chain production is an important modifier of β-thalassemia disease. This including the nature of β-thalassemia mutation, number of functional α-globin genes and genetic factor that regulate HbF production^2^. Our two previous genome-wide SNPs association studies (GWAS) had been performed in order to search for genetic modifying factors in β^0^-thalassemia/HbE disease^3,4^. The GWAS analysis identified a number of highly significant SNPs located in 3 regions on chromosome 2p16.1, 6q23.3 and 11p15.4. The strongest association with the disease severity is located in the region of more than 200 kb harboring several genes in the β-globin cluster and nearby olfactory receptor gene on chromosome 11p15.4. The second most significantly associated region mapped to the *HBS1L*-*MYB* intergenic 24 kb region on chromosome 6q23.3. The third significantly disease severity associated SNPs are located in the *BCL11A* gene on chromosome 2p16.1.

Discovery of genetic modifiers for β^0^-thalassemia/HbE provided a genetic basis for clinical application such as new targets for therapeutic intervention and predicting disease severity. Predictive SNPs for β^0^-thalassemia/HbE disease severity have a wide range of clinical applications, such as a better informed genetic counseling, clinical management and assistance in therapeutic decisions. However, as several SNPs demonstrated to be disease modifiers, single SNP might not be a good prediction of disease severity. In addition, genetic factors modify disease through a complex mechanism of multiple genes interaction of a biological network. Here the two β^0^-thalassemia/HbE GWAS data^3,4^ of 487 cases were reanalyzed to search for minimum predictive SNPs for disease severity by using a SNP interaction prioritization algorithm, interacting Loci (iLoci)^5^. Combination of three SNPs located on chromosome 2p16.1 (*BCL11A*), 6q23.3 *(HBS1L*-*MYB)* and 11p15.4 (*HBE1*) could predict disease severity with more than 85% specificity and 75% accuracy. The three predictive SNPs were validated in two independent cohorts of Thai and Malaysian patients with similar accuracy and specificity.

## Results

### Characteristics of cohort study

Characteristics of the patients according to demographic information and hematological data are described in Table S1. Types of β-thalassemia mutations in this study are illustrated in Table S2. The most frequent β-thalassemia mutations in Thai β^0^-thalassemia/HbE patients were HBB:c.126-129delCTTT and HBB:c.52A>T in the cohort 1 (45.7% and 26.3%, respectively) and the cohort 2 (51.6% and 23.4%, respectively). While the cohort 3, Malaysian patients, HBB:c.92 + 5G>C (37.9%) and HBB:c.92 + 1G>A (22.4%) were the most frequent mutations.

### Identification of predictive SNPs

As multiple SNPs demonstrated to be disease modifiers of β^0^-thalassemia/HbE, single SNP might not have enough power to predict disease severity. Thus, reanalysis of SNP-SNP interaction of the previously reported two GWAS data^3,4^ was performed by iLOCi algorithm^5^. The highest disease severity associated SNP pair ranked by the SNP pair interaction analysis was rs766432/rs9399137, which predicts disease severity at 62.5% accuracy (Table 1). Combination of the two highest disease severity associated SNP pairs, rs766432/rs9399137 and rs72872548/rs9399137 could increase prediction accuracy to 72.0%. While three SNP pairs, combination of the prior two pairs and rs11208724/rs1407273 have 72.3% prediction accuracy (Table 1). Although the disease severity prediction accuracy increased with the increasing number of SNP pairs, the prediction accuracy of two or more SNP pairs was not much different. Thus, only three SNPs from two SNP pairs were selected for further evaluation, which are rs766432 located in *BCL11*A gene on chromosome 2p16.1, rs9399137 located in *HBS1L-MYB* intergenic region on chromosome 6q23.3 and rs72872548, located in *HBE1* gene on chromosome 11p15.4.

**Table 1:** SNPs pairs interaction analysis of the GWAS data by iLOCi algorithm searching for predictive SNPs for disease severity of β^0^-thalassemia/HbE patients.

Number of SNP pairs	SNP pairs				Total SNPs	% Accuracy
	I	II	III	IV		
1	rs766432 (*BCL11A*) rs9399137 (*HBS1L-MYB)*				2	62.5
2	rs766432 (*BCL11A*) rs9399137 (*HBS1L-MYB*)	rs72872548 (*HBE1*) rs9399137 (*HBS1L-MYB*)			3	72.0
3	rs766432 (*BCL11A*) rs9399137 (*HBS1L-MYB*)	rs72872548 (*HBE1*) rs9399137 (*HBS1L-MYB*)	rs11208724 (*LEPR*) rs1407273 (*HMCN1*)		5	72.3
4	rs766432 (*BCL11A*) rs9399137 (*HBS1L-MYB*)	rs72872548 (*HBE1*) rs9399137 (*HBS1L-MYB*)	rs11208724 (*LEPR*) rs1407273 (*HMCN1*)	rs7584288 (*THUMPD2*) rs3814161 (*GDF10*)	7	73.0

In addition, reanalysis for single SNP association of the disease severity was performed on the two GWAS data by FaST-LMM, which utilizes the linear mixed model to select the associated SNPs^6^. The three SNPs, rs766432 (*BCL11A*), rs9399137 *(HBS1L*-*MYB)* and rs72872548 (*HBE1*), showed high level of significance among the three previously reported regions that strongly associated with disease severity (chromosome 2p16.1, chromosome 6q23.3 and chromosome 11p15.4)^3,4^ (Table 2). The agreement between two reanalysis methods suggested that the three SNPs were good candidate predictive SNPs for constructing the disease severity predictive model. The allele frequency of the three predictive SNPs in cohort 1 Thai β^0^-thalassemia/HbE patients compared with another ethnic group is showed in Table S3.

**Table 2:** Single SNPs analysis of the GWAS data by FaST linear mixed models for SNPs associated with disease severity in β^0^-thalassemia/HbE patients.

SNP ID	Chromosome	Position^a^	*P*-value
**rs766432**	2	**60492835**	**2.25 × 10**^**–10**^
rs6729815	2	60496537	3.01 × 10^–9^
rs6545817	2	60488044	4.18 × 10^–9^
rs6545816	2	60487726	1.15 × 10^–8^
rs17331129	2	60452807	1.59 × 10^–7^
rs7775698	6	135097497	4.99 × 10^–11^
**rs9399137**	6	**135097880**	**5.30 × 10**^**–11**^
rs9376092	6	135106006	2.24 × 10^–10^
rs9494145	6	135111414	2.45 × 10^–10^
rs4895441	6	135105435	3.90 × 10^–10^
**rs72872548**	**11**	**5267909**	**7.46 × 10**^**–4**^
rs969258452	11	5261221	7.53 × 10^–4^
b-thal2855036*	11	5251444	2.75 × 10^–3^
bg51*	11	5236565	3.37 × 10^–3^
rs7482144 (*Xmn*I)	11	5254939	4.50 × 10^–3^

The three predictive SNPs are indicated by bold letter.

^a^Nucleotide positions for the SNPs are relative to the GenBank accession number GRCh38.p12 * = SNP not in NCBI database.

### Determination of risk score for β-thalassemia/HbE disease severity predictive SNPs

β^0^-Thalassemia/HbE patients have a wide spectrum of clinical symptoms. To determine whether the selected SNPs account for moderate symptom patients, the three predictive SNPs were genotype in 181 patients with moderate symptom as well as in the mild and severe symptoms in the discovery cohort 1. The genotype frequency of the three predictive SNPs among 668 β^0^-thalassemia/HbE patients in the cohort 1 with different severity (mild, moderate and severe) is showed in Table 3. Odds ratio (OR) analysis was then performed to determine risk genotypes of each SNP between two different disease severity groups; mild vs moderate, mild vs severe and moderate vs severe in the cohort 1 (Table 4). The rs766432 genotype AA has increased risk for severe symptoms (mild vs severe: OR = 0.39, 95% CI = 0.26–0.58, *P* = 4.98 × 10^–6^; moderate vs severe: OR = 0.51, 95% CI = 0.34–0.77, *P* = 1.45 × 10^–3^). The rs9399137 genotype TT has increased risk for severe symptoms (mild vs severe: OR = 0.35, 95% CI = 0.23–0.51, *P* = 2.16 × 10^–7^). While the rs72872548 genotype CC has increased risk for severe symptoms (mild vs severe: OR = 0.21, 95% CI = 0.12–0.36, *P* = 2.25 × 10^–9^; moderate vs severe: OR = 0.50, 95% CI = 0.32–0.77, *P* = 1.98 × 10^–3^).

**Table 3:** Genotype frequencies of predictive SNPs among mild, moderate and severe β^0^-thalassemia/HbE patients in cohort 1.

SNP ID	Genotype	Disease severity		
		Mild (n = 180)	Moderate (n = 181)	Severe (n = 307)
rs766432 (*BCL11A*)	CC	3.89	4.42	1.95
	AC	37.22	30.39	19.54
	AA	58.89	65.19	78.50
	Total	100.00	100.00	100.00
rs9399137 (*HBS1L-MYB)*	CC	2.78	1.66	0.33
	TC	40.00	23.20	20.20
	TT	57.22	75.14	79.48
	Total	100.00	100.00	100.00
rs72872548 (*HBE1*)	AA	11.67	1.66	1.63
	AC	78.33	77.35	63.84
	CC	10.00	20.99	34.53
	Total	100.00	100.00	100.00

**Table 4:** The risk genotypes of the predictive SNP among mild, moderate and severe β^0^-thalassemia/HbE patients in cohort 1.

SNP ID	Genotypes	Mild vs moderate			Mild vs severe			Moderate vs severe		
		Odd ratio	95% CI	*P-*value	Odd ratio	95% CI	*P-*value	Odd ratio	95% CI	*P-*value
rs766432 (*BCL11A*)	AA	0.76	0.49–1.17	0.23	**0.39**	**0.26–0.58**	**4.98 × 10**^**–6**^	**0.51**	**0.34–0.77**	**1.45 × 10**^**–3**^
	AC	1.36	0.87–2.10	0.18	**2.44**	**1.61–3.69**	**2.09 × 10**^**–5**^	**1.80**	**1.17–2.74**	**7.95 × 10**^**–3**^
	CC	0.88	0.31–2.46	1.00	2.03	0.67–6.13	0.24	2.32	0.79–6.79	0.15
rs9399137 (*HBS1L-MYB)*	TT	**0.44**	**0.28–0.69**	**3.63 × 10**^**–4**^	**0.35**	**0.23–0.51**	**2.16 × 10**^**–7**^	0.78	0.50–1.20	0.31
	TC	**2.21**	**1.39–3.48**	**6.73 × 10**^**–4**^	**2.63**	**1.75–3.96**	**3.35 × 10**^**–6**^	1.19	0.76–1.86	0.49
	CC	1.70	0.39–7.20	0.50	**8.74**	**1.01–75.43**	**2.78 × 10**^**–2**^	5.16	0.53–49.95	0.14
rs72872548 (*HBE1*)	AA	**7.84**	**2.29–26.77**	**9.36 × 10**^**–5**^	**7.98**	**2.95–21.55**	**3.51 × 10**^**–6**^	1.02	0.24–4.31	1.00
	AC	1.06	0.64–1.74	0.89	**2.05**	**1.33–3.12**	**1.09 × 10**^**–3**^	**1.93**	**1.27–2.93**	**2.33 × 10**^**–3**^
	CC	**0.42**	**0.22–0.76**	**5.40 × 10**^**–3**^	**0.21**	**0.12–0.36**	**2.25 × 10**^**–9**^	**0.50**	**0.32–0.77**	**1.98 × 10**^**–3**^

Bold letter indicate *P*-value < 0.01.

In order to apply the risk genotypes for disease severity prediction, score was assigned to the SNP genotypes as 0, 1 and 2 accordingly to the risk from low to high risk of severe symptom. Therefore, SNPs predictive risk score for rs766432 were CC = 0, AC = 1 and AA = 2; for rs9399137 were CC = 0, TC = 1 and TT = 2 and for rs72872548 were AA = 0, AC = 1 and CC = 2 (Table 5). The score of each predictive SNPs was combined and used for interpretation of disease severity prediction. Three scoring models of interpretation of the combine SNPs risk score were generated (Table 5) and evaluated in the 668 β^0^-thalassemia/HbE cohort 1. The first scoring model, model 1, yield the highest sensitivity, 60.0%, and accuracy, 71.2%. While the model 3 resulted in the highest specificity, 81.4% (Table 6). To determine which scoring model is better in disease severity prediction, the three scoring models were further validated in two independent Thai and Malaysian β^0^-thalassemia/HbE cohorts.

**Table 5:** Predictive SNP scoring system for β^0^-thalassemia/HbE disease severity.

SNP ID	Gene	SNPs score	
rs766432	*BCL11A*	CC = 0, AC = 1, AA = 2	
rs9399137	*HBS1L-MYB*	CC = 0, TC = 1, TT = 2	
rs72872548	*HBE1*	AA = 0, AC = 1, CC = 2	

Scoring model	Mild	Moderate	Severe
Model 1	0–3	4–5	5–6
Model 2	0–3	4	5–6
Model 3	0–3	4–5	6

**Table 6:** Sensitivity, specificity and accuracy of predictive SNP score system assessed from cohort patients.

Scoring model	Cohort	Sensitivity	Specificity	Accuracy
Model 1	Cohort 1	60.0	76.7	71.2
	Cohort 2	65.6	80.5	75.5
	Cohort 3	70.7	84.5	79.9
Model 2	Cohort 1	47.9	80.0	69.3
	Cohort 2	43.8	82.8	69.8
	Cohort 3	53. 5	85.3	74.7
Model 3	Cohort 1	37.0	81.4	66.6
	Cohort 2	56.3	85.2	75.5
	Cohort 3	50.0	88.8	75.9

### Validation of predictive SNP scoring system

The three-SNP risk scoring models were validated in 122 cases β^0^-thalassemia/HbE patients in two cohorts, 64 Thai patients (cohort 2) and 58 Malaysian patients (cohort 3). The allele frequency of the three predictive SNPs in the Thai and Malaysian validation cohorts are shown in Table S3. Consistence with the results from cohort 1, the model 1 have the highest sensitivity and accuracy compared to model 2 and model 3 in every cohorts. While the model 3 have highest specificity in every cohorts (Table 6).

## Discussion

β^0^-Thalassemia/HbE patients have a wild range of disease severity from the unrequired of regular blood transfusion to transfusion dependent. Prediction of the disease severity is helpful for clinical management and quality of life of the patients. Knowing the severity of the affected child during pregnancy could aid in genetic counseling. After birth, this could also be useful for clinical decisions of transfusion program such as frequency and age needed for blood transfusion. In addition, this will aid in deciding whether to perform hematopoietic stem cell transplantation, which is more efficient if it is performed early in life. Nevertheless, clinical phenotype can take few years to stabilize, and in such a situation the possibility of anticipating the clinical severity could be essential. Two β^0^-thalassemia/HbE GWAS studies have identified a number of risk SNPs. Here, FaST-LMM, iLOCi and machine learning software by WEKA was used to examine SNPs interaction and determine predictive SNP risk score.

This study explored the predictive SNPs for disease severity in 668 β^0^-thalassemia/HbE patients. Analysis of SNP interaction revealed that rs766432 (*BCL11A*), rs9399137 (*HBS1L-MYB*) and rs72872548 (*HBE1*) are highest associated with clinical symptoms. Three risk scoring models, which differ in assigning score 5 to moderate and/or severe symptoms, were evaluate for prediction of clinical severity in three independent cohorts. This due to the rs72872548 genotype AC is high frequency among mild, moderate and severe cases, 78.33, 77.35 and 63.84%, respectively. The score model 1 have the highest sensitivity and accuracy. However, it would difficult to predict whether the patients would be moderate or severe for those who have risk score 5. The score model 3 have highest specificity, 81.4–88.8%, in every cohorts. Although, assigning score 5 to moderate group might have some severe patient miss prediction as moderate, this rather better than predict moderate patients as severe patient in such cases the patients might undergo unnecessary aggressive treatments.

The three predictive SNPs identified here are located in three loci that associated with HbF levels, which affect the central mechanism underlying disease pathophysiology, the degree of the excess of α-globin chains and globin chain imbalance. Several GWAS studies showed the three loci are associated with HbF level^3,7,8^. The *BCL11A* is a transcription factor act as the specific erythroid repressor of HbF expression in the developmental silencing of the mouse and human *HBG* genes^9,10^. The *HBS1L-MYB* intergenic region contains distal enhancers required for *MYB* activation^11^. The c-MYB, a transcription factor, is key regulator of hematopoiesis and erythropoiesis. cMYB represses *HBG* genes expression via KLF1 activation of BCL11A^12^. In addition, several GWAS studies showed association of SNPs on *BCL11A* and *HBS1L-MYB* with red blood cells parameters such as mean corpuscular volume (MCV)^13,14^. The β-globin cluster, *Xmn*1-*HBG2* (rs782144) influence on HbF level has long been discovered through candidate and genetic linkage studies and later confirmed by GWAS studies. The rs72872548 (*HBE1*) is located in the same haplotype block as the *Xmn*1-*HBG2.*

The use of multiple genetic factors to predict β-thalassemia was first reported in a study in Sardinian homozygous β^0^-thalassemia. Three genetic factors, α-thalassemia, rs11886868 in *BCL11A* and rs9389268 in *HBS1L-MYB* were account for 75% of the phenotype severity^15^. A study of a mix of Mediterranean (3/4) and Asian (1/4) patients from France used five genetic factors, β-thalassemia mutations, α-thalassemia, the *Xmn*1-*HBG2*, rs11886868 in *BCL11A* and rs9399137 in *HBSB1L-MYB* have 83.2% predictive accuracy^16^. A thalassemia severity score (TSS) was developed from 890 homozygous β-thalassemia patients of the Mediterranean basin using these different genetic modifiers including sex, β-thalassemia mutations, α-thalassemia, the *Xmn*1-*HBG2*, rs1427407 and rs1018957 in *BCL11A* and rs9399137 in *HBS1L-MYB*^17^. In this study, the β^0^-thalassemia/HbE patients who carry β^+^-thalassemia mutations or were found positive for the common Thai α-thalassemia mutations were excluded. Hence, the predictive score comprise of only three SNPs, which are located at the same loci as the previous studied.

The limitation of the predictive SNPs scoring is that different ethnic groups may have different allele frequency and linkage disequilibrium structures. This might render the scoring less accurate in untested populations. The TSS score was validated in a North African cohort which showed that allelic frequencies of the SNPs are different compared to the Mediterranean^18^. According to the International HapMap Project, the rs1018957 (*BCL11A*) allele A/G frequency are quite different among European (0.580/0.420), African (0.722/0.278) and this study Thai β^0^-Thalassemia/HbE patients (0.182/0.818). The allele frequency of the predictive SNPs in this study of both Thai and Malaysian population are comparable. This suggested that the three SNPs can be used as predictive SNPs for disease severity at least for Southeast Asia where β^0^-thalassemia/HbE is prevalent.

This study, to the best of our knowledge, is the first SNP risk score for prediction of clinical severity developed for a β^0^-thalassemia/HbE Southeast Asia population. This may assist in inform prognosis and guide therapeutics. However, the predictive SNPs of this study might not predict disease severity in β^+(severe)/^β^0^-thalassemia and β^0^/β^0^-thalassemia patients who a have higher degree of α- to non-α-globin imbalance and being the cause of the severe thalassemia phenotype. Nevertheless, the predictive SNPs validation also required in other populations.

## Methods

### Subjects and thalassemia diagnosis

This study was performed in accordance with the Helsinki Declaration and was approved by the Mahidol University Institutional Review Board, Nakhon Pathom, Thailand (approval number MU-IRB 2007-083, 2008/303.2001, 2009/128.2306, 2010/010.0701 and 2012/035.2802) and The Ethical Review Committee of Pathology Department, Hospital Sultanah Bahiyah, Kedah, Malaysia (approval number NMRR-12-980-13829 (IIR)). Written informed consent was obtained from all individual participants included in the study.

Three cohorts of β^0^-thalassemia/HbE patients were enrolled, Thai discovery cohort (cohort 1), Thai validation cohort (cohort 2) and Malaysian validation cohort (cohort 3). In this study, the β^0^-thalassemia/HbE patients who carry β^+^-thalassemia mutations or were found positive for the common Thai α-thalassemia mutations were excluded. The cohort 1 comprised 668 cases (180 mild, 181 moderate and 307 severe) of Thai β^0^-thalassemia/HbE patients. A total of 487 cases (180 mild and 307 severe) were randomly selected from the two GWAS analysis of mild and severe cases^3,4^. In addition 181 moderate patients were newly combining in the cohort 1. Two independent validation cohorts of Thai and Malaysian patients comprised 64 Thai cases and 58 Malaysian cases, respectively.

After informed consent was obtained, EDTA blood was collected and hematological data analysis was performed using an automated cell counter (ADVIA 120, Bayer, Tarrytown, NY). Hemoglobin analysis and quantification were determined by the automated hemoglobin cation exchange high-performance liquid chromatography (Bio-Rad variant II, Bio-Rad Hercules, CA). The β-thalassemia mutations were characterized using reverse dot blot hybridization^19^. The α-thalassemia deletional mutations were genotyped by multiplex GAP-PCR^20^. The α-thalassemia point mutations, Hb Constant Spring and Hb Pakse, were determined by dot blot hybridization^21^. The disease severity of β^0^-thalassemis/HbE presenting for mild, moderate and severe symptoms was classified by scoring system based on 6 parameters; hemoglobin at steady state, age at receiving first blood transfusion, requirement for blood transfusion, size of spleen, age at thalassemia presentation and the growth and development^22^.

### Predictive SNP discovery

The disease severity association analysis was performed on the two separated GWAS analysis datasets^3,4^ of 180 mild and 307 severe of cohort 1 by two techniques i.e. iLOCi^5^ for SNP interaction and FaST-LMM^6^ for single SNP association. The predictive model was later constructed using a standard machine learning software, Waikato Environment for Knowledge Analysis or WEKA (http://www.cs.waikato.ac.nz/ml/weka/). The classification accuracy of disease severity was measured from the Hidden Naive Bayes model included in WEKA.

### Genotyping of predictive SNPs

Genomic DNA was extracted from peripheral blood using the Gentra Puregene Blood Kit (Qiagen, Hilden, Germany). The three predictive SNPs; rs766432, rs9399137 and rs72872548, located on chromosome 2p16.1 (*BCL11A*), 6q23.3 *(HBS1L-MYB)* and 11p15.4 (*HBE1*), respectively, were genotyped by high-resolution melting (HRM) analysis using primers indicated in Table S4 and SsoFast EvaGreen Supermix (Bio-Rad, Hercules, CA) on CFX96 real-time PCR system (Bio-Rad).

### Statistical analysis

All statistical procedures were performed using SPSS v.18.0 software package (SPSS Inc., Chicago, IL). Odds ratio (OR) analysis was used to identify risk genotypes of the predictive SNPs associated with disease severity.

## Supplementary Information

Supplementary Information.
