# Supplementary material for: Predictive SNPs for β0-thalassemia/HbE disease severity
Source: Sci Rep. 2021 May 14;11:10352. doi: 10.1038/s41598-021-89641-2 (PMC8121782; doi:10.1038/s41598-021-89641-2)
Supplement: Supplementary file 1 — Supplementary Information. [file 41598_2021_89641_MOESM1_ESM.pdf]

Supplementary information

Predictive SNPs for β^0-thalassemia/HbE disease severity

Thongperm Munkongdee[1,2], Sissades Tongsima[3], Chumpol Ngamphiw[3], Pongsakorn Wangkumhang[3], Chayanon Peerapittayamongkol[1], Hafizah Binti Hashim[4], Suthat Fucharoen[1,2], Saovaros Svasti[2, 5,]*

[1]Department of Biochemistry, Faculty of Medicine Siriraj Hospital, Mahidol University, Bangkok, Thailand; [2]Thalassemia Research Center, Institute of Molecular Biosciences, Mahidol University, Nakhon Pathom, Thailand; [3] National Biobank of Thailand (NBT), National Science and Technology Development Agency (NSTDA), Pathum Thani, Thailand; [4]Pathology Department, Hospital Sultanah Bahiyah, Kedah, Malaysia; [5]Department of Biochemistry, Faculty of Science, Mahidol University, Bangkok, Thailand.

Number of supplement table: 4

[*] Corresponding author:

Saovaros Svasti, Ph.D.

Thalassemia Research Center, Institute of Molecular Biosciences, Mahidol University

25/25 Phuttamonthon 4 Road, Salaya, Nakhon Pathom 73170 Thailand.

Tel: +66-2889-2557, Fax: +66-2889-2559 ORCID: 0000-0001-8334-9838

E-mail: saovaros.sva@mahidol.ac.th, stssv@yahoo.com

Table S1. Hematological parameters of the three cohorts

	Cohort 1 Thai (discovery)			Cohort 2 Thai Thai (validation)			Cohort 3 Malaysian (validation)		
	**Mild**	**Moderate**	**Severe**	**Mild**	**Moderate**	**Severe**	**Mild**	**Moderate**	**Severe**
**No. (%)**	180	181	307	15	32	17	2	34	22
**Gender, male (%)**	40	45.3	55.3	40	46	64.7	100	47	60
**Age at presentation (years)**	7.0 ± 8.4	4.2 ± 4.0	1.6 ± 1.4	6.9 ± 6.3	3.4 ± 2.8	1.9 ± 1.4	5, 20	6.0 ± 8.9	2.9 ± 2.1
**Age at receiving first transfusion (years)**	13.4 ± 9.0	8.2 ± 8.6	2.7 ± 2.5	16.7 ± 14.9	7.0 ± 9.6	4.6 ± 2.0	20	7.9 ± 8.6	3.1 ± 2.4
**Requriement for regular transfusion (%)**	2 (1.1)	76 (41.9)	289 (94.1)	0 (0)	13 (40)	17 (100)	0 (0)	28 (82.3)	22 (100)
**Never requriement for regular transfusion (%)**	92 (51.1)	3 (1.65)	0 (0)	6 (40)	2 (6.25)	0 (0)	1 (50)	0 (0)	0 (0)
**Spleen size < 3 cm (%)**	57 (31.6)	26 (14.3)	5 (1.6)	15 (100)	4 (12.5)	1 (5.8)	2 (100)	5 (14.7)	3 (13.6)
**Splenectomy (%)**	4 (2.2)	57 (31.5)	178 (58.0)	0 (0)	5 (15.6)	1 (5.8)	0 (0)	3 (8.8)	4 (18.1)
**Normal growth development (%)**	118 (65.5)	90 (49.7)	64 (20.8)	15 (100)	23 (71.8)	4 (23.5)	2 (100)	21 (61.7)	11 (50)
**Hemoglobin level (g/dL)***	7.7 ± 1.3	5.8 ± 1.5	4.5 ± 1.4	8.2 ± 1.3	6.3 ± 1.4	4.9 ± 1.3	7. 2, 8.3	6.6 ± 2.8	2.3 ± 1.5
**Hb F (%)**	41.6 ± 11.2	35.0 ± 10.9	31.1 ± 11.9	41.4 ± 12.9	37.7 ± 12.1	34.2 ± 12.7	46.8, 55.8	43.9 ± 17.5	30.7 ± 15.8
**Hb F (g/dL)**	3.18 ± 1.1	2.0 ± 0.8	1.4 ± 0.7	3.5 ± 1.5	2.5 ± 1.2	1.7 ± 0.8	4.0 , 4.0	3.1 ± 1.9	0.8 ± 0.9

***Hemoglobin levels were obtained from the average of hemoglobin levels at steady state or before receiving blood transfusion.**

Table S2. Frequency of β-thalassemia mutation in the three β^0-thalassemia/HbE cohorts

Mutations	HGV Name	**Cohort 1** Thai (discovery)		**Cohort 2** Thai (validation)		**Cohort 3** Malaysian (validation)	
		Number of cases	Frequency	Number of cases	Frequency	Number of cases	Frequency
Int (ATG>ACG)	HBB:c.2T>C	1	0.1				
Cod 15 (-T)	HBB:c.46delT	2	0.3				
Cod 15 (G>A)	HBB:c.47G>A	1	0.1				
Cod 17 (A>T)	HBB:c.52A>T	176	26.3	15	23.4	2	3.4
Cod 27/28 (+C)	HBB:c.84_85insC	4	0.6				
IVS I-1 (G>A)	HBB:c.92+1G>A	1	0.1				
IVS I-1 (G>T)	HBB:c.92+1G>T	24	3.6	4	6.3	13	22.4
IVS I-2 (T>C)	HBB:c.92+2T>C					1	1.7
IVS I-5 (G>C)	HBB:c.92+5G>C	45	6.7	4	6.3	22	37.9
IVS I (25 bp deletion)	HBB:c.93-21_96del					2	3.4
Cod 35 (C>A)	HBB:c.108C>A	8	1.2				
Cod 35 (-C)	HBB:c.108delC					2	3.4
Cod 41 (-C)	HBB:c.126delC	1	0.1				
Cod 41/42 (-TTCT)	HBB:c.126_129delCTTT	305	45.7	33	51.6	10	17.2
Cod 43 (G>T)	HBB:c.130G>T	3	0.4				
Cod 71/72 (+A)	HBB:c.216_217insA	20	3.0	5	7.8		
Cod 95 (+A)	HBB:c.287_288insA	1	0.1				
Cod 98 (T>G)	HBB:c.296T>G					2	3.4
IVS II-654 (C>T)	HBB:c.316-197C>T	67	10.0	3	4.7	2	3.4
Cod 110 (T>C)	HBB:c.332T>C	1	0.1				
Cod 123/124/125	HBB:c.370	4	0.6			2	3.4
3.4 kb deletion		4	0.6				
**Total**		**668**	**100.0**	**64**	**100.0**	**58**	**100.0**

Table S3. Allele frequency of the three predictive SNPs for disease severity in Thai, Malaysian and other populations

	**rs766432 (*BCL11A*)**		**rs9399137 (*HBS1L-MYB*)**		**rs72872548 (*HBE1*)**	
**Chromosome (Position[a])**	**2 (60492835)**		**6 (135097880)**		**11 (5267909)**	
	**Ref Allele C**	**Alt allele A**	**Ref Allele T**	**Alt allele C**	**Ref Allele C**	**Alt allele A**
**Cohort 1: Thai (discovery)[b]**	0.168	0.832	0.855	0.145	0.600	0.400
**Cohort 2: Thai (validation)[b]**	0.227	0.773	0.891	0.109	0.570	0.430
**Cohort 3: Malaysian (validation)[b]**	0.095	0.905	0.871	0.129	0.491	0.509
**East Asian[c]**	0.261	0.739	0.756	0.244	0.873	0.127
**South Asian[c]**	0.130	0.870	0.890	0.110	0.730	0.270
**African[c]**	0.269	0.731	0.958	0.042	0.719	0.281
**Europe[c]**	0.154	0.846	0.738	0.262	0.698	0.302
**American[c]**	0.230	0.770	0.850	0.150	0.810	0.190

Ref Allele, reference allele; Alt allele, alternative allele.

[a] Nucleotide positions for the SNPs are relative to the GenBank accession number GRCh38.p12

[b] β^0-thalassemia/HbE patients in this study

[c] Sample population of the International HapMap Project

Table S4. List of primer for the predictive SNPs genotyping

SNP ID	Sequence (5′ to 3′)	Amplicon size (bp)
rs766432-F	CGC TTT AGC TTT ATT AAG GTA	80
rs766432-R	AGT TGG ATT CAA TTT GCC AG	
rs9399137-F	GGG ATG TAA TTA ACT GAA CAT AT	48
rs9399137-R	GGG TTG CTT GTG AAA AAA CTG	
rs72872548-F	GAG CAG GCT ATT GTT GAT AC	76
rs72872548-R	GAA GTA GCT AGC TTG TCA CAT	
